# Chronic Health Conditions as a Risk Factor for Falls among the Community-Dwelling US Older Adults: A Zero-Inflated Regression Modeling Approach

**DOI:** 10.1155/2017/5146378

**Published:** 2017-03-28

**Authors:** Yoshita Paliwal, Patricia W. Slattum, Scott M. Ratliff

**Affiliations:** ^1^Department of Pharmacotherapy and Outcomes Science, School of Pharmacy, Virginia Commonwealth University, Richmond, VA 23298, USA; ^2^Division of Epidemiology, Virginia Commonwealth University, Richmond, VA 23219, USA

## Abstract

Falls are an important health concern among older adults due to age-related changes in the body. Having a medical history of chronic health condition may pose even higher risk of falling. Only few studies have assessed a number of chronic health conditions as risk factor for falls over a large nationally representative sample of US older adults. In this study, Behavioral Risk Factor Surveillance System (BRFSS) 2014 participants aged 65 years and older (*n* = 159,336) were evaluated. It was found that 29.7% (*n* = 44,550) of the sample experienced at least one fall and 16.3% (*n* = 20,444) experienced more than one fall in the past 12 months. According to the study findings, having a medical history of stroke, CKD, arthritis, depression, and diabetes independently predict the risk of first-time falling as well as the risk of recurrent falling in older adult population while controlling for other factors. On the other hand, having a medical history of the heart attack, angina, asthma, and COPD did not predict the risk of first-time falling, but did predict the risk of recurrent falling after experiencing the first fall in this population.

## 1. Background

Falls and associated health consequences are significant public health issues among older adults. Nearly 30% of older adult population experience a fall incident every year [1]. According to the Center for Disease Control and Prevention (CDC), nearly 6 million US older adults experienced at least one fall, and nearly 2 million suffered from at least one fall-related injury in 2006 [2]. Falls also put major social, psychological, and financial burdens on the victims and their caregivers [1].

Many factors may contribute to falls. Literature identifies various biological, social, environmental, and behavioral risk factors for falls among different populations and age groups [1–3]. Among biological risk factors, the age, race, and gender of a person and a history of chronic health conditions (CHCs) may play an important role to predict falls. Previous studies report various CHCs as a risk factor for falling. For example, evidences for arthritis as a risk factor for falls are well documented in the literature [4–6]. Studies show a positive association between chronic obstructive pulmonary disease (COPD) and the risk of falls [7, 8]. Studies also report an increased risk of falling among patients with a medical history of stroke [9, 10]. Many studies identify depression as a major risk factor for falls [11–13]. However, studies in the past have been limited to assessing only one health condition at a time and often used a small set of sample population. It is important to understand that older adults often suffer from multiple health conditions [14]. Therefore, assessing only one particular health condition as a risk factor for falls may challenge the accuracy of study findings.

Also, using logistic regression to evaluate episodic data such as falls where many of the study participants do not experience a fall during the evaluation period does not capture the overdispersion due to excessive zeroes in the data. Thus, the results may be misleading. The zero-inflated regression modeling approach can address this issue. Zero-inflated regression models include both logistic and Poisson components, which allow for the identification of distinct predictors for the first-time (nonrecurrent) falling and recurrent falling, while accounting for excessive zeroes in the data at the same time. Previous studies repeatedly used the traditional method of logistic regression modeling, and, therefore, the risk of falling recurrently after the first event of falling could not be addressed.

The primary objective of this study was to quantify the current prevalence of falls, recurrent falls, and a number of CHCs in US older adult population. The secondary objective of this study was to examine those CHCs as risk factors for falls and recurrent falls in this population. This study also evaluated various demographic, socioeconomic, and behavioral risk factors as potential confounders for establishing the true relationship between CHCs and falls. Understanding the relationship between CHCs and falls is important to inform public health programs to increase the awareness regarding falls and CHCs as risk factors and to guide the development of interventions to decrease the risk of falls while addressing CHCs.

## 2. Methodology

### 2.1. Data Source and Sample Population

We performed a secondary data analysis using the Behavioral Risk Factor Surveillance System (BRFSS) survey 2014. BRFSS is a large cross-sectional US population based survey. It is conducted by the CDC every year in all the 50 states of US as well as the District of Columbia and three US territories [15]. BRFSS gathers information on various sociodemographic, behavioral, and health-related factors, as well as healthcare and preventive care utilization. BRFSS uses a multistage sampling design based on the random digit dialing methods to collect data from the community-dwelling US population. Details of the study design, implementation, study measures, and survey procedures of BRFSS have been described previously [16].

### 2.2. Sample Population

A sample of 464,664 individuals, who participated in the BRFSS 2014, was restricted to the older adults aged 65 years and older (*n* = 159,336). Of those 159,336 older adults, 149,876 answered the question about falls.

### 2.3. Study Outcome and Predictors

The main outcome variable in this study was self-reported “falls” in the previous 12 months in response to the following question: “The next question asks about a recent fall. By a fall, we mean when a person unintentionally comes to rest on the ground or another lower level. In the past 12 months, how many times have you fallen?” [15] We considered falls as a categorical variable for descriptive and logistic regression analyses and as a count variable for zero-inflated regression analyses.

We examined a number of CHCs as explanatory variables in this study in response to the survey question: “Have you ever been diagnosed by this chronic condition?” Participants were asked about ten chronic health conditions in BRFSS including the heart attack, angina, stroke, asthma, cancer, COPD, CKD, arthritis, depression, and diabetes [15]. We considered participants as having a medical history of a CHC if they responded “yes” to the question about that CHC.

Other than the CHCs as predictors, we included demographic (age, sex, race, and marital status), socioeconomic (education, employment, and income), and behavioral (smoking and drinking) covariates in our study. We identified these covariates as potential confounders as being associated with the risk of falls in previous literature. We considered two categories of age, “65–79” and “80+,” as the risk of falling increases substantially in people aged 80 and above [17]. We categorized education as “less than or equal to a high school education” and “more than high school education.” Rationale for this categorization was based on the findings from previous studies where fall rates reportedly decreased among people with the higher level of education [18, 19]. We divided income into two major categories of “less than $50,000” and “equal to or more than $50,000.” This division was based on the evidences in the literature where a low income was associated with an increased risk of falls [20, 21].

We created two categories for employment. One for the people who are currently engaged in some kind of work or work-related activity named as “employed” and another category for people who are retired or unable to work named as “unemployed.” Unemployment may pose a higher risk of falling indirectly due to lack of access to the social and healthcare [22]. We categorized marital status into two groups, “married or living with a partner” and “single or unmarried.” Living alone may pose a significant risk for falling recurrently followed by the first episode of fall, considering the absence of a support system in the family [23]. We included sex (grouped into male and female) and race (grouped into whites, black, Hispanics, and others) as it is as categorized into BRFSS 2014 [15]. We also included smoking and drinking in our analysis as previous literature reports an association between these behavioral factors and falls [24].

### 2.4. Statistical Analyses and Model Building

We used SAS version 9.4 for the descriptive and the inferential analyses. A *p* value of less than or equal to 0.05 was considered significant for all analyses for statistical decision-making. We generated weighted prevalence estimates and proportional differences between characteristics of fallers and nonfallers using chi-square tests in the descriptive analysis.  Table 1 summarizes the findings from descriptive analysis. Further, we generated weighted unadjusted and adjusted odds ratios representing the likelihood of falls among older adults with a number of CHCs using logistic regression analyses.  Table 2 shows findings from the logistic regression analyses.

Since 70.3% (*n* = 105,326) participants answered zero to the question on the frequency of falling in the past 12 months, we identified the data as overdispersed due to the presence of excess zeroes. To address this issue of overdispersion, we conducted zero-inflated regression modeling. In contrast to the simple logistic or Poisson model, zero-inflated count models assume that the data consists of two different groups, zero and nonzero. The zero group represents nonfallers (who did not yet experience a fall) and the nonzero group represents fallers (who already experienced at least one fall event) [25]. We estimated the risk of first-time falling (or nonrecurrent falling) among nonfallers and the risk of recurrent falling among fallers. The logit part of the zero-inflated model shows the likelihood of being in the zero group (nonfallers) by generating odds ratio estimates. Predictors with lower odds of being in this group will have higher odds of experiencing a fall for the first time and being transferred to the nonzero group (fallers). The count part of the zero-inflated model shows the risk of experiencing recurrent falls among the nonzero group (fallers) by generating relative risk estimates.  Table 3 provides the logit and count part estimates from both ZIP and ZINB models.

We further compared both model fits by AIC (Akaike Information Criterion), BIC (Bayesian Information Criterion), and standard errors of their estimates. Compared to ZIP model, most ZINB model parameters were found to be smaller in magnitude with larger standard errors. Both ZIP and ZINB accounted for excess zeroes, yet the Pearson statistic of ZIP model is indicated for the model misspecification, and in that case the observed standard errors may be biased. Therefore, we considered the ZINB model to be a better fit to explain CHCs as risk factors for falling for the first time and then recurrently among the community-dwelling US older population.  Table 4 provides a comparison of the model fit criteria for two models.

## 3. Results

### 3.1. Characteristics of Participants

This study includes 159,336 community-dwelling older adults who participated in the 2014 BRFSS. Of these, 29.7% (*n* = 44,500) older adults experienced at least one fall, and 16.3% (*n* = 20,444) experienced more than one fall in the past 12 months. Among the participants, 43.8% were male and 56.2% were female. Of these, 77.5% were 65–79 years old, and 22.5% were 80 years and older. The majority of the participants were Caucasians (79.6%), followed by African-American (8.7%), Hispanics (7.6%), and others (4.2%). Approximately, 54% of participants had a medical history of arthritis, 23% had a history of diabetes, 17% had a history of cancer, 13% had a history of angina, 12% had a history of asthma, 12% had a history of heart attack, 8% had a history of stroke, and 6% had a history of CKD (Table 1).

### 3.2. Risk Association between CHCs and Falls

#### 3.2.1. Logistic Regression Analyses

While assessing the relationship between CHCs and falls with logistic regression analyses, we found angina, stroke, asthma, cancer, CKD, arthritis, depression, and diabetes as significant predictors for falls, while controlling for other sociodemographic and behavioral factors. A summary of the bivariate and multivariate logistic regression analyses is presented in Table 2. We found a positive association between fall and angina (OR: 1.17, 95% CI: 1.09–1.27), stroke (OR: 1.61, 95% CI: 1.46–1.76), asthma (OR: 1.22, 95% CI: 1.13–1.32), cancer (OR: 1.13, 95% CI: 1.06–1.20), CKD (OR: 1.27, 95% CI: 1.14–1.42), arthritis (OR: 1.61, 95% CI: 1.52–1.70) depression (OR: 2.26, 95% CI: 2.11–2.42), and diabetes (OR: 1.32, 95% CI: 1.24–1.40). Among sociodemographic factors, age, gender, marital status, income, and employment were statistically significant predictors of falls. Being in the older (80+) age group, having low income, being white, female, unmarried, or single, and being unemployed significantly increase the odds of falling among older adults (Table 2). Among behavioral factors, heavy drinkers showed higher odds of having a fall compared to their counterparts, adjusting for other sociodemographic predictors and CHCs (Table 2).

#### 3.2.2. Zero-Inflated Regression Analyses

The logit part of the ZINB model addresses the significant predictors for membership between the zero group (nonfallers) and nonzero group (fallers). Older adults with a medical history of stroke, cancer, CKD, arthritis, depression, and diabetes had significantly lower odds of being in the zero group (nonfallers). Hence, they will have higher odds of experiencing a fall for the first time and moving into the nonzero group (fallers). Older adults with the depression had the lowest odds of being in the zero group (nonfallers), and thus they showed the highest risk of experiencing a fall event. Among sociodemographic factors, age, gender, race, marital status, and employment were significant predictors for membership between zero and nonzero group. Older adults who were 80 years or older, white, female, and those who were unmarried/single and unemployed had lower odds of being in the zero group and hence a higher risk of experiencing a fall. Also, being a smoker increases the odds of experiencing a fall event.

The count part of the ZINB model addresses the relative risk of experiencing recurrent falls among nonzero group (fallers) who already experienced a fall. We found a positive association between the number of fall episodes and the heart attack (RR: 1.16, 95% CI: 1.11–1.21), angina (RR: 1.17, 95% CI: 1.12–1.22), stroke (RR: 1.50, 95% CI: 1.43–1.57), asthma (RR: 1.22, 95% CI: 1.18–1.27), COPD (RR: 1.13 95% CI: 1.08–1.17), CKD (RR: 1.24, 95% CI: 1.18–1.31), arthritis (RR: 1.39, 95% CI: 1.34–1.44), depression (RR: 2.12, 95% CI: 2.05–2.20), and diabetes (RR: 1.18, 95% CI: 1.14–1.22) among fallers. In sociodemographic characteristics, being a male and white, being in the younger (65–79) age group, and being unmarried or single significantly increase the number of fall episodes after experiencing the first fall among fallers. Also, being unemployed, having lower income, and being a smoker significantly increase the risk of recurrent falls after the first event.

Summarizing the findings from this most appropriately fit ZINB model, we observed that having a medical history of stroke, CKD, arthritis, depression, and diabetes independently predict the risk of first-time falling as well as the risk of recurrent falling in older adult population while controlling for other factors. On the other hand, having a medical history of the heart attack, angina, asthma, and COPD did not predict the risk of first-time falling but did predict the risk of recurrent falling after experiencing the first fall in this population. Cancer was observed as the only health condition which predicted the risk of first-time falling but did not predict the risk of recurrent falling.

## 4. Discussion

This study evaluates a number of CHCs as risk factor of falling for the first time and recurrently among a large and nationally representative sample of community-dwelling US older adults. We used the recently available BRFSS 2014 dataset for the analysis which includes a number of CHCs which has been identified as the most leading causes of mortality and morbidity in the US by CDC [26]. So, the results of this study are more generalizable and represent community-dwelling US older adults at large. Also, reliability and the validity of BRFSS have been examined in various studies and found comparable or greater as compared to other national surveys, so using BRFSS for the analysis strengthens the internal validity of this study [16]. Another strength of this study is the inclusion and evaluation of a number of major CHCs against falls, instead of a particular health condition. Older adults often suffer from more than one health condition at the same time; and the number of comorbidities increases with the age [14]. Previous literature has been limited to the assessment of only one specific condition at a time using a small sample population. Including a number of CHCs in our analysis, we observed the absolute risk associated with a particular CHC while adjusting for others. This way we increased the accuracy and robustness of our findings.

Methodologically, we used the zero-inflated regression modeling in addition to traditional logistic modeling, which is another major strength of this study. Most previous studies used logistic regression analysis to evaluate CHCs as risk factors for falls. However, fall data often consists of excess zeroes with a high proportion reporting no falls, and, due to this issue of overdispersion, results may be misleading. Literature reports that first-time fallers and recurrent fallers possess distinct characteristics and hence are two different populations [20, 21]. However, this has not been evaluated among older adult populations with CHCs. We observed a distinction between various CHCs as how they predict the risk of experiencing a first fall and then a risk of having recurrent fall episodes. It is important to address the risk for both populations distinctly as risk stratification and fall risk reduction interventions may need to be different for nonrecurrent fallers compared to recurrent fallers.

Often studies on falls use different time frames and different age groups, so it is challenging to compare our findings with previous studies. Compared to a recent study in Canadian older adults, the prevalence of falls reported in our study (29.7%) is larger than the prevalence reported (20%) in this study [17]. However, both studies show a positive association between a number of CHCs and falls [17]. Our prevalence findings, otherwise, are similar to few studies reported for the same population age and time frame of one year [18, 19].

An important finding in our study is the strong positive association between falls and depression. Both multivariate logistic regression and zero-inflated regression predict more than a twofold increased risk of falling among older adults with depression. Both parts of ZINB identify the depression as risk factor for fall, which means depression increases the risk of falling for the first time as well as falling recurrently among older adults. This supports previous findings on depression as a risk factor for falls and also adds to the evidence by adjusting for other CHCs and documenting the risk associated with the first fall as well as recurrent falls. Healthcare providers and researchers should pay particular attention to depressive symptoms while developing and implementing fall interventions, as depression shows the strongest relationship with the risk of fall among this population. Following depression, arthritis and stroke were found to be major risk factors for falls in our analysis. These chronic health conditions should particularly be targeted while developing fall assessments and interventions. Current fall prevention guidelines recommend a multifactorial fall assessment only when a person fails the test of postural balance [27]. Chronic conditions such as depression, diabetes, COPD, and CKD do not directly impact the postural balance, yet they show a significant association with falls. Older adults with these CHCs should also be considered for a comprehensive assessment of fall risk. Further, regular health checkups can identify a CHC in the early stages for potentially treating and educating patients about these CHCs to potentially reduce further risk of falling. Our study informs public health researchers about two different “at-risk” populations: first fallers and recurrent fallers. Targeting these two populations separately with collaborative prevention and disease management strategies at individual, clinical and community level could be an efficient fall reduction approach.

Our study has some limitations as well. BRFSS includes land-line phones only, so there could be a selection bias while choosing the sample population. Also, BRFSS does not include older adults residing in the long-term care facilities/nursing homes; hence our results are not generalizable to the institutionalized older adult population. Recalling a fall event in the past 12 months may be challenging for some older adults, resulting in the underestimation of the prevalence of falls, so there could be a recall bias. Also, there is a possibility of information bias as participants might not report information accurately (e.g., diagnosis of the CHCs) which may lead to misclassification resulting in under- or overestimation of risk.

Some age-related health conditions, such as neurodegenerative diseases, poor vision, gait, and balance disorders that increase the risk for falling, were not investigated in BRFSS 2014. Some other factors which have been identified previously as important predictors for falls, such as previous falls, medications, and other environmental factors, were also not captured in BRFSS 2014 [28]. Not including these factors and health conditions in our analyses may potentially confound our results. For example, medications prescribed for the CHCs might be responsible for the falls in addition to the risk associated with the condition itself. It may also be that falls are associated with poorly controlled CHCs rather than simply the presence of the condition, but this cannot be assessed in this study. Overall, BRFSS is a nationally representative survey with an established reliability and validity [16]. So the results of this study are generalizable to a large set of community-dwelling US older adults.

## 5. Conclusion

While falls remain a common health issue among older adults, this study concludes that there are two different “at-risk” populations. One group includes older adults who are at increased risk of experiencing a fall. This group includes people who are older (over 80 years of age), female, white, single/unmarried, employed, and diagnosed with the stroke, cancer, CKD, arthritis, depression, or diabetes. The other group includes older adults who are at increased risk of experiencing recurrent fall episodes after falling for the first time. This group includes people who are younger (65–79), male, white, and diagnosed with the heart attack, angina, stroke asthma, COPD, CKD, arthritis, depression, and diabetes. These groups may benefit from different risk stratification and interventions. Though demographic factors cannot be modified, CHCs can still be managed to reduce falls and related consequences. Fall prevention guidelines can be improved by recommending comprehensive fall assessment among older adults with CHCs who are significant predictors of fall.

## Figures and Tables

**Table 1:** Characteristics of community-dwelling US older adults by fall, BRFSS 2014.

Characteristics	Overall sample	Fallers^a^ *N* (weighted%)	Nonfallers *N* (weighted%)	*χ* ^2^ *p* value
*Age group*				
65–79	113747	32335 (27.2%)	81412 (72.8%)	<0.0001
80+	36129	12215 (33.5%)	23914 (66.5%)	

*Race *				
White	128911	38878 (29.5%)	90033 (70.5%)	<0.0001
Black	8870	2250 (23.3%)	6620 (76.7%)	
Hispanic	6196	1687 (26.2%)	4509 (73.8%)	
Other	5899	1735 (26.5%)	4164 (73.5%)	

*Gender *				
Male	56726	15842 (26.5%)	40884 (73.5%)	<0.0001
Female	93150	28708 (30.3%)	64442 (69.7%)	

*Marital status *				
Married/living-in	73368	19806 (26.2%)	53562 (73.8%)	<0.0001
Unmarried/single	75900	24587 (31.7%)	51313 (68.3%)	

*Education *				
≤High school education	61842	18136 (28.2%)	43706 (71.8%)	0.0771
>High school education	87568	26284 (29.0%)	61284 (71.0%)	

*Employment *				
Employed	25181	6477 (23.9%)	18704 (76.1%)	<0.0001
Unemployed	124076	37895 (29.5%)	86181 (70.5%)	

*Income *				
<$50,000/Year	80873	25528 (30.5%)	55345 (69.5%)	<0.0001
≥$50,000/Year	41008	10949 (25.3%)	30059 (74.7%)	

*Binge drinking* ^b^				
Yes	5845	1835 (31.3%)	4010 (68.7%)	0.0248
No	141342	41982 (28.6%)	99360 (71.4%)	

*Smoking*				
Yes	12561	3748 (28.6%)	8813 (71.4%)	0.9896
No	136200	40480 (28.6%)	95720 (71.4%)	

*Ever had heart attack*				
Yes	16947	6383 (36.1%)	10564 (63.9%)	<0.0001
No	131974	37796 (27.6%)	94178 (72.4%)	

*Ever had angina*				
Yes	17712	6662 (36.0%)	11050 (64.0%)	<0.0001
No	129887	37034 (27.4%)	92853 (72.6%)	

*Ever had stroke*				
Yes	11131	4790 (42.5%)	6341 (57.5%)	<0.0001
No	138208	39542 (27.5%)	98666 (72.5%)	

*Ever had asthma*				
Yes	17439	6501 (36.0%)	10938 (64.0%)	<0.0001
No	131948	37860 (27.6%)	94088 (72.4%)	

*Ever had cancer*				
Yes	26061	8591 (31.9%)	17470 (68.1%)	<0.0001
No	123428	35838 (27.9%)	87590 (72.1%)	

*Ever had COPD* ^c^				
Yes	18301	6781 (36.3%)	11520 (63.7%)	<0.0001
No	130525	37344 (27.4%)	93181 (72.6%)	

*Ever had CKD* ^d^				
Yes	8649	3552 (40.9%)	5097 (59.1%)	<0.0001
No	140558	40722 (27.7%)	99836 (72.3%)	

*Ever had arthritis*				
Yes	80538	28533 (34.4%)	52005 (65.6%)	<0.0001
No	68519	15730 (21.7%)	52789 (78.3%)	

*Ever had depression*				
Yes	23670	11174 (47.3%)	12496 (52.7%)	<0.0001
No	125631	33155 (25.2%)	92476 (74.8%)	

*Ever had diabetes*				
Yes	31716	11279 (34.3%)	20437 (65.7%)	<0.0001
No	117956	33210 (26.9%)	84746 (73.1%)	

*Note*. Data source: 2014 BRFSS (Behavioral Risk Factor Surveillance System). Results are weighted estimates.

^a^Fallers = those who had experienced at least one fall in past 12 months.

^b^Binge drinking = males having five or more drinks on one occasion and females having four or more drinks on one occasion.

^c^COPD = Chronic Obstructive Pulmonary Disorder.

^d^CKD = Chronic Kidney Condition.

**Table 2:** Risk factors of falls among community-dwelling US older adults: logistic regression modeling, BRFSS 2014.

Explanatory variables (reference)	Crude odds ratio (95% CI)	Standard error	Adjusted odds ratio (95% CI)	Standard error
*Chronic health conditions*				
Heart attack	1.48 (1.39–1.58)	0.0332	—	—
Angina	1.49 (1.40–1.59)	0.0329	1.17 (1.09–1.27)	0.0389
Stroke	1.96 (1.81–2.11)	0.0396	1.61 (1.46–1.76)	0.0474
Asthma	1.48 (1.38–1.58)	0.0342	1.22 (1.13–1.32)	0.0399
Cancer	1.21 (1.14–1.28)	0.0301	1.13 (1.06–1.20)	0.0329
COPD^a^	1.51 (1.42–1.61)	0.0323	—	—
CKD^b^	1.81 (1.63–1.99)	0.0515	1.27 (1.14–1.42)	0.0553
Arthritis	1.89 (1.80–1.98)	0.0237	1.61 (1.52–1.70)	0.0277
Depression	2.67 (2.52–2.83)	0.0296	2.26 (2.11–2.42)	0.0350
Diabetes	1.42 (1.34–1.49)	0.0268	1.32 (1.24–1.40)	0.0320

*Sociodemographic composition*				
Age group 80+ (65–79)	1.35 (1.28–1.42)	0.0263	1.27 (1.20–1.36)	0.0330
Black (White)	0.73 (0.66–0.80)	0.0482	0.68 (0.60–0.75)	0.0567
Hispanic (White)	0.85 (0.75–0.96)	0.0622	0.74 (0.65–0.85)	0.0701
Other race (White)	0.86 (0.71–1.04)	0.0961	0.80 (0.66–0.97)	0.0982
Female (male)	1.21 (1.15–1.26)	0.0234	1.06 (1.00–1.12)	0.0284
Unmarried/single (married/living-in)	1.31 (1.25–1.37)	0.0231	1.13 (1.07–1.20)	0.0293
Unemployed (employed)	1.33 (1.25–1.42)	0.0325	1.10 (1.02–1.18)	0.0370
Income <$50,000/year (≥$50,000/year)	1.30 (1.23–1.37)	0.0268	1.07 (1.00–1.13)	0.0305
Binge drinking^c^ (no binge drinking)	1.14 (1.02–1.27)	0.0571	1.24 (1.09–1.41)	0.0654

*Note*. Data source: 2014 BRFSS (Behavioral Risk Factor Surveillance System).

^a^COPD = Chronic Obstructive Pulmonary Disorder.

^b^CKD = Chronic Kidney Condition.

^c^Binge drinking = males having five or more drinks on one occasion and females having four or more drinks on one occasion.

CI = Confidence Interval.

**Table 3:** Risk factors of falls among community-dwelling US older adults: zero-inflated regression modeling, BRFSS 2014.

Explanatory variables (reference)	Zero-inflated Poisson		Zero-inflated negative binomial	
Count part	RR (95% CI)	Standard error	RR (95% CI)	Standard error

*Chronic health conditions*				
Heart attack	1.13 (1.10–1.16)	0.0122	1.16 (1.11–1.21)	0.0219
Angina	1.08 (1.05–1.10)	0.0118	1.17 (1.12–1.22)	0.0209
Stroke	1.32 (1.29–1.35)	0.0115	1.50 (1.43–1.57)	0.0249
Asthma	1.15 (1.12–1.17)	0.0113	1.22 (1.18–1.27)	0.0201
COPD^a^	1.10 (1.08–1.13)	0.0110	1.13 (1.08–1.17)	0.0203
CKD^b^	1.18 (1.15–1.21)	0.0132	1.24 (1.18–1.31)	0.0276
Arthritis	1.17 (1.15–1.19)	0.0095	1.39 (1.34–1.44)	0.0176
Depression	1.68 (1.65–1.71)	0.0089	2.12 (2.05–2.20)	0.0186
Diabetes	1.11 (1.09–1.13)	0.0093	1.18 (1.14–1.22)	0.0177

*Sociodemographic composition*				
Age group 80+ (65–79)	0.89 (0.87–0.90)	0.0108	0.94 (0.91–0.98)	0.0191
Black (White)	0.74 (0.71–0.77)	0.0223	0.70 (0.65–0.75)	0.0355
Hispanic (White)	0.89 (0.85–0.93)	0.0231	0.85 (0.78–0.92)	0.0401
Other race (White)	1.10 (1.06–1.14)	0.0190	1.09 (1.01–1.18)	0.0394
Female (male)	0.76 (0.74–0.77)	0.0090	0.72 (0.70–0.75)	0.0184
Unmarried/single (married/living-in)	0.98 (0.96–0.99)	0.0093	1.06 (1.03–1.10)	0.0169
Unemployed (employed)	1.11 (1.08–1.14)	0.0120	1.14 (1.09–1.19)	0.0216
Income <$50,000/year (≥$50,000/year)	1.17 (1.14–1.19)	0.0105	1.11 (1.08–1.14)	0.0154
Smoking (no smoking)	1.22 (1.19–1.25)	0.0130	1.13 (1.07–1.19)	0.0281

Logit part	OR (95% CI)	Standard error	OR (95% CI)	Standard error

*Chronic health conditions*				
Heart attack	0.92 (0.88–0.97)	0.0253	—	—
Angina	0.90 (0.86–0.94)	0.0244	—	—
Stroke	0.71 (0.68–0.75)	0.0267	0.44 (0.30–0.64)	0.1903
Asthma	0.88 (0.85–0.93)	0.0224	—	—
Cancer^a^	0.88 (0.84–0.92)	0.0190	0.64 (0.53–0.79)	0.1015
CKD^b^	0.84 (0.79–0.89)	0.0298	0.56 (0.36–0.87)	0.2216
Arthritis	0.65 (0.63–0.68)	0.0161	0.37 (0.31–0.44)	0.0901
Depression	0.55 (0.53–0.58)	0.0189	0.22 (0.14–0.33)	0.2171
Diabetes	0.83 (0.79–0.85)	0.0184	0.60 (0.50–0.72)	0.0999

*Sociodemographic composition*				
Age group 80+ (65–79)	0.74 (0.71–0.77)	0.0198	0.14 (0.07–0.26)	0.3274
Black (White)	1.21 (1.12–1.30)	0.0370	1.56 (1.14–2.14)	0.1618
Hispanic (White)	1.19 (1.10–1.29)	0.0401	1.58 (1.16–2.15)	0.1579
Other race (White)	1.13 (1.05–1.22)	0.0377	1.73 (1.31–2.29)	0.1431
Female (male)	0.83 (0.80–0.86)	0.0165	0.33 (0.27–0.40)	0.1059
Unmarried/single (married/living-in)	0.86 (0.83–0.89)	0.0170	0.75 (0.64–0.88)	0.0827
Unemployed (employed)	—	—	1.34 (1.11–1.62)	0.0950
Income <$50,000/year (≥$50,000/year)	1.06 (1.03–1.10)	0.0181	—	—
Smoking (no smoking)	1.15 (1.03–1.10)	0.0258	1.68 (1.38–2.06)	0.1030

*Note*. Data source: 2014 BRFSS (Behavioral Risk Factor Surveillance System).

^a^COPD = Chronic Obstructive Pulmonary Disorder.

^b^CKD = Chronic Kidney Condition.

CI = Confidence Interval.

RR = relative risk; OR = odds ratio.

**Table 4:** Comparison of model fit criteria of ZIP and ZINB models.

Criterion	ZIP^a^	ZINB^b^
Scaled Pearson *χ*^2^	3.4216	2.4309
Full log likelihood	−144381.3621	−118910.3002
AIC^c^	288836.7242	237890.6003
BIC^d^	289194.1387	238228.6952

*Note*. ^a^ZIP = zero-inflated Poisson.

^b^ZINB = zero-inflated negative binomial.

^c^AIC = Akaike Information Criterion.

^d^BIC = Bayesian Information Criterion.
